# Hydrothermal Synthesis of Ultrasmall Pt Nanoparticles as Highly Active Electrocatalysts for Methanol Oxidation

**DOI:** 10.3390/nano5042203

**Published:** 2015-12-08

**Authors:** Wenhai Ji, Weihong Qi, Shasha Tang, Hongcheng Peng, Siqi Li

**Affiliations:** 1 School of Materials Science and Engineering, Central South University, Changsha 410083, China; E-Mails: jwh1988@csu.edu.cn (W.J.); tang0360@ntu.edu.sg (S.T.); 133111028@csu.edu.cn (H.P.); 9901090160@csu.edu.cn (S.L.); 2 Institute for Materials Microstructure, Central South University, Changsha 410083, China; 3 Key Laboratory of Non-Ferrous Materials Science and Engineering, Ministry of Education, Changsha 410083, China

**Keywords:** hydrothermal method, ultrasmall nanoparticles, PVP, methanol oxidation

## Abstract

Ultrasmall nanoparticles, with sizes in the 1–3 nm range, exhibit unique properties distinct from those of free molecules and larger-sized nanoparticles. Demonstrating that the hydrothermal method can serve as a facile method for the synthesis of platinum nanoparticles, we successfully synthesized ultrasmall Pt nanoparticles with an average size of 2.45 nm, with the aid of poly(vinyl pyrrolidone) (PVP) as reducing agents and capping agents. Because of the size effect, these ultrasmall Pt nanoparticles exhibit a high activity toward the methanol oxidation reaction.

## 1. Introduction

Platinum has been widely used in many applications, especially for catalysis and fuel cell technology, due to its ability to facilitate both oxidation and reduction reactions [1,2,3,4,5]. Size and shape play an important role in determining properties and potential applications of nanomaterials. Thus, downsizing these nanomaterials can provide a great opportunity to achieve a high surface-to-volume ratio and thus enhance the metal utilization in noble metal–based catalysts or electrocatalysts. Ultrasmall nanoparticles (USNPs), with sizes in the 1–3 nm range, exhibit unique properties distinct from those of free molecules and larger-sized nanoparticles.

Pt USNPs have also been reported. By using alcohols as reductants in the presence of poly(vinyl pyrrolidone) (PVP) in a refluxing aqueous system, Pt USNPs were obtained [6]. The average size of Pt USNPs could be controlled from 1.9 to 3.3 nm by changing the alcohol or the concentrations of the reagents. Photochemical methodology also could be used as a very effective way to produce Pt USNPs [7]. It involved visible light as a reaction trigger and platinum acetylacetonate and thioglycolic acid as the only chemical reactants. The average diameter of platinum nanoparticles was 1.0 ± 0.3 nm. Li *et al.* reported the synthesis of monodisperse Pt USNPs stabilized with peptides in aqueous solution at room temperature [8]. The specifically selected peptide molecule, P7A, is able to bind to the surface of the Pt NPs and regulate the nucleation and growth rates, affording monodisperse Pt NPs with sizes in the 1.7–3.5 nm range. Pt USNPs have also been synthesized with hydrophobic ligands. Uniform 2-nm-sized Pt USNPs were obtained by the decomposition of platinum dibenzylideneacetone under mild conditions in the presence of n-octylsilane [9]. Lim *et al.* successfully synthesized Pt USNPs with an average size of 1.9 nm through the reaction of a Pt salt precursor with water, without the aid of any exotic reducing agents and organic capping molecules [10]. The *in situ* synthesis of Pt USNPs at room temperature using poly(4-vinyl phenol) (PVPh) as both the reducing as well as the stabilizing agent in aqueous alkaline solution has been reported [11]. Transmission electron microscopic analysis confirms the formation of ultra-small spherical Pt USNPs from 1.6 to 2.2 nm in diameter with a high degree of monodispersity depending on the ratio of PVPh to platinum salt concentrations used in a single reaction.

**Scheme 1:**
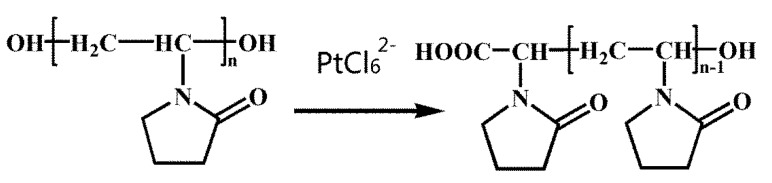
Formulas of OH-terminated poly(vinyl pyrrolidone) (PVP) and the structural variation in the effect of PtCl_6_^2−^ ions.

Specific organic capping agents or stabilizers have been exploited in order to restrict nanocrystal growth as well as to provide a barrier to agglomeration. Notably, PVP has received special attention due to its high chemical stability, nontoxicity, and excellent solubility in many polar solvents. Hydroxyl end groups of PVP can be applied as a general strategy for the kinetically controlled synthesis of nanoplates made of noble metals such as Ag, Pd, Au, and Pt [12,13]. PVP (see Scheme 1) has an affinity for long-chain alcohols owing to its hydroxyl end groups; it can serve as a reducing agent. As a major advantage over alcohols with short alkyl chains commonly used in chemical synthesis, the PVP can be used as both reductant and stabilizer.

The hydrothermal method has been used in the synthesis of semiconductor nanocrystals and nanocomposites for a long time, but its role remains largely unexplored in the synthesis of noble-metal colloids. Here we demonstrate that Pt USNPs can be synthesized through a hydrothermal method with PVP. To the best of our knowledge, the present work may be the first report on synthesizing Pt USNPs in aqueous solutions by means of a simple hydrothermal method.

## 2. Results and Discussion

**Figure 1:**
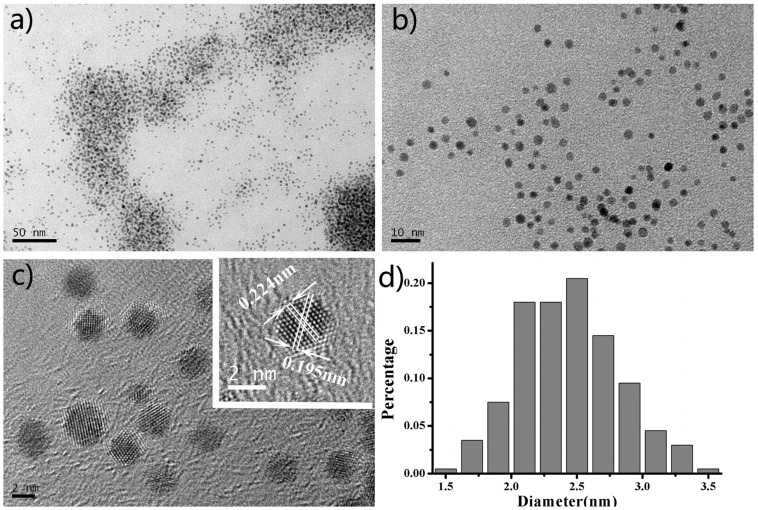
(**a**,**b**) Typical transmission electron microscope (TEM) images of ultrasmall Pt nanoparticles at different magnification and (**c**,**d**) high-resolution TEM image and the corresponding size distributions of the samples.

**Figure 2:**
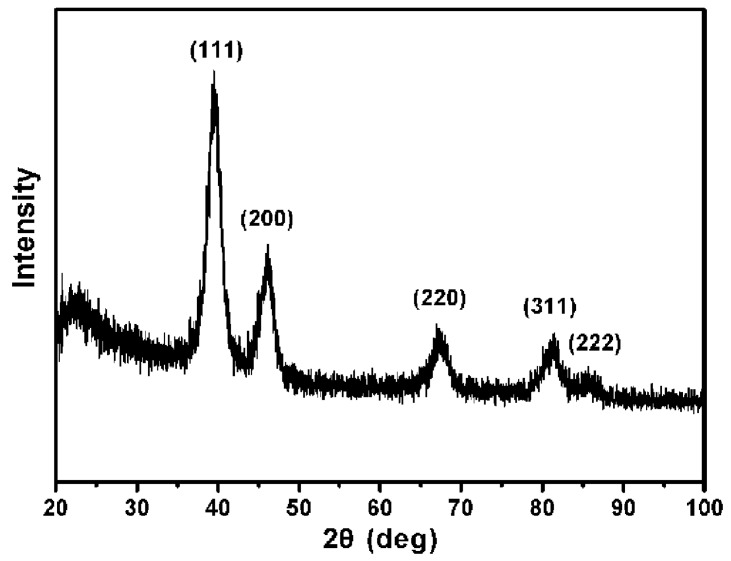
Powder X-Ray diffraction (XRD) pattern of ultrasmall Pt nanoparticles.

We synthesized Pt USNPs by reacting K_2_PtCl_6_ with PVP in a closed autoclave in an oven at 160 °C for 8 h. Transmission electron microscope (TEM) images in Figure 1a,b show that the Pt USNPs have an average size around 2.45 nm. The high resolution TEM image of the crystal structure of the Pt USNPs in Figure 1c displays a well-defined crystal lattice. The inset image in Figure 1c shows that a d spacing between adjacent lattice planes of 0.224 nm corresponds to the {111} planes, and 0.195 nm corresponds to the {200} planes. TEM studies and a size histogram of Pt USNPs obtained by counting 200 particles (Figure 1d) show that these Pt USNPs typically have nearly spherical shapes, with a narrow size distribution. The formation of Pt USNPs synthesized in the hydrothermal method is due to the presence of PVP. The OH-terminated PVP can be applied as the reductant for the synthesis of nanoparticles made of other noble metals (such as Ag, Pd, Au, and Pt) [12,13,14,15]. Compared with alcohols (commonly used in the chemical synthesis), the PVP can be used as both a reductant and stabilizer. Scheme 1 shows the formula of OH-terminated PVP and the structural changes effected by PtCl_6_^2−^ ions. The powder X-Ray diffraction (XRD) pattern of the product (Figure 2) was matched with that of bulk Pt (Fm-3m, a = 3.924, PDF No. 89–7382), confirming the formation of metallic Pt USNPs with a face-centered cubic structure.

**Figure 3:**
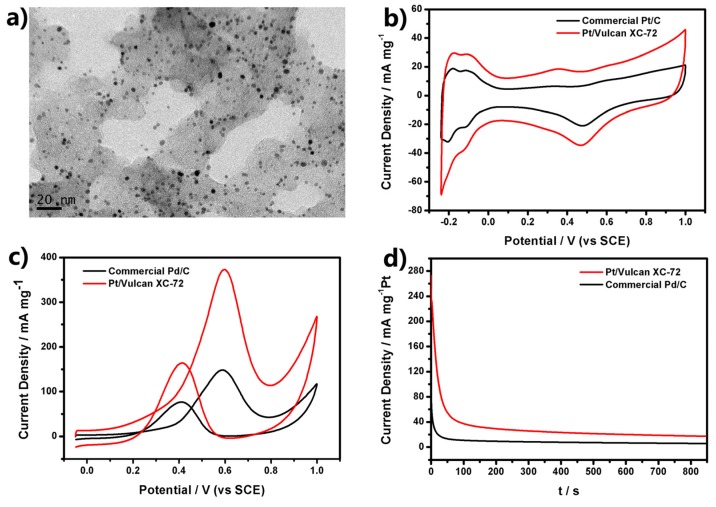
(**a**) TEM images of Pt ultrasmall nanoparticles (USNPs) supported on Vulcan XC-72 carbon; (**b**) Cyclic voltammetry (CV) curves of Pt electrocatalysts in N_2_-saturated 0.5 M H_2_SO_4_ with a scan rate of 50 mV·s^−1^; (**c**) CV curves at a scan rate of 50 mV·s^−1^ with different Pt electrocatalysts in 0.5 M H_2_SO_4_ + 0.5 M CH_3_OH; (**d**) Amperometric *i*-*t* curves of Pt electrocatalysts in 0.5 M H_2_SO_4_ + 0.5 M CH_3_OH solution at 0.6 V (*vs.* saturated calomel electrode (SCE)).

We tested the electrochemical behaviors of the prepared Pt USNPs. In order to minimize possible aggregation during the catalytic reaction and utilize the support effect, we used Vulcan XC-72 as the support. Vulcan XC-72 carbon, which is the most widely used support material for direct methanol fuel cell electrodes, is amorphous with a specific surface area of 212 m^2^·g^−1^. A given weight of Vulcan XC-72 was mixed with the as-prepared solution of Pt USNPs for an hour. The products were separated and dried in a vacuum oven. Figure 3a shows that a number of Pt USNPs were distributed almost evenly over the entire surfaces of the Vulcan carbon powders. We measured cyclic voltammetry (CV) curves of Pt/Vulcan XC-72 catalysts in N_2_-saturated 0.5 M H_2_SO_4_ in comparison with the commercial Pt/C catalyst (E-TEK, 30% Pt on Vulcan XC-72 carbon support). CV curves of Pt/Vulcan XC-72 catalysts (Figure 3b) reveal a large peak between −0.24 and 0.08 V (*vs.* saturated calomel electrode (SCE)), which is associated with the hydrogen adsorption/desorption processes. The electrochemically active surface area (ECSA) can be calculated from the charge involved in the hydrogen adsorption/desorption processes using Equation (1) [16].

(1) $$ECSA=Q/\left[Pt\right]\times 0.21\text{ mC}\cdot {\text{cm}}^{-2}$$

where *Q* (mC) and [Pt] are the charge for hydrogen adsorption/desorption and the loading of Pt on the electrode, respectively, while 0.21 mC·cm^−2^ is the electrical charge associated with the monolayer adsorption of hydrogen on Pt [17]. The ECSA of the Pt/Vulcan XC-72 is as high as 46.2 m^2^/g_Pt_, which is 1.52 times greater than that of the commercial Pt/C catalyst (30.3 m^2^/g_Pt_). It demonstrated Pt/Vulcan XC-72 catalysts had much higher ECSA.

The electrochemical performance of the Pt/Vulcan XC-72 composite (shown in Figure 3a) was tested for methanol oxidation, which is at the heart of direct methanol fuel cells (DMFC) application in the anodic half-cell reaction. For comparison, the commercial Pt/C catalyst was also evaluated in the same condition. The electrocatalytic performances of the Pt-based electrocatalysts towards methanol oxidation were evaluated using CV and amperometric *i*-*t* in 0.5 M H_2_SO_4_ + 0.5 M CH_3_OH solution. CV curves in Figure 3c show two prominent peaks in both positive and negative scans corresponding to the methanol oxidation for all tested catalysts, and it can be seen that the current from methanol oxidation becomes apparent as the potential rises to above 0.2 V. In the forward scan, methanol oxidation produces an anodic peak at around 0.6 V. In the reverse scan, an anodic peak appears at around 0.4 V. This anodic peak in the reverse scan can be attributed to the removal of the incompletely oxidized carbonaceous species formed in the forward scan. These features of CV curves are in agreement with the reports for the Pt/C catalyst. As shown in Figure 3c, the peak current of methanol oxidation on Pt/Vulcan XC-72 is 373 mA·mg^−1^ Pt, which is much higher than that of the commercial Pt/C catalyst (148.3 mA·mg^−1^ Pt). The ratio of the forward oxidation current peak (*I*_f_) to the reverse current peak (*I*_b_), *I*_f_/*I*_b_, is an index of the catalyst tolerance to the poisoning species. A higher ratio indicates more effective removal of the poisoning species on the catalyst surface. The *I*_f_/*I*_b_ ratio of Pt/Vulcan XC-72 is 2.27, higher than that of the commercial Pt/C (1.93), showing better catalyst tolerance of the Pt/Vulcan XC-72 catalyst.

To further compare the electrochemical long-term performance between Pt/Vulcan XC-72 and commercial Pt/C, a chronoamperometry test was conducted in the solution of 0.5 M H_2_SO_4_ and 0.5 M CH_3_OH for 850 s at a fixed potential of 0.6 V (*vs.* SCE), as shown in Figure 3d. The initial high current density was attributed to the double-layer charging and the numerous available active sites of Pt atoms. The currents of all the catalysts decreased rapidly within first few seconds due to the formation of CO-like intermediates such as CO and CHO, *etc.* [18,19,20,21], which generated during the oxidation of methanol and adsorbed on the surface of active Pt atoms to prevent methanol’s further oxidation on these Pt atoms. After that, the currents kept on decreasing and gradually maintained a steady state. The current density of Pt/Vulcan XC-72 was higher than that of commercial Pt/C during the whole testing time. The current density of Pt/Vulcan XC-72 is 17.2 mA·mg^−1^ at 850 s, which is much higher than that of commercial Pt/C (5.6 mA·mg^−1^ at 850 s). The higher current density directly reflected the higher resistivity of Pt/Vulcan XC-72 to the poisoning of CO-like intermediates.

## 3. Experimental Section

### 3.1. Chemicals and Materials

Potassium hexachloroplatinate (IV) (K_2_PtCl_6_, AR) and methanol were purchased from Shanghai Chemical Reagent Company (Shanghai, China). Poly(vinyl pyrrolidone) (PVP, *M*_w_ = 55,000) was received from Aldrich. Vulcan XC-72 was purchased from Cabot (Boston, MA, USA) and 5% Nafion was purchased from Sigma (Shanghai, China). All chemicals were used as received without further purification. High-purity deionized water (>18.4 MΩ·cm) was produced using Millipore A10 Milli-Q (Darmstadt, Germany).

### 3.2. Synthesis of Pt USNPs

A typical synthesis process can be concisely described as follows. K_2_PtCl_6_ (38.8 mg) and PVP (44.4 mg, *M*_w_ = 55,000) were added into water (10 mL), and the solution was stirred for 10 min. Then the homogeneous orange-yellow solution was transferred to a 15 mL Teflon-lined stainless steel autoclave. The container was then sealed in a stainless steel bomb. The whole system was heated and maintained in an oven at 160 °C under autogenous pressure for 8 h. After the reaction finished, the container was cooled under room temperature conditions naturally. Finally, a solution of Pt particles with dark gray color was obtained.

### 3.3 Synthesis of Pt USNPs/Vulcan XC-72

A given weight of Vulcan XC-72 was mixed with the as-prepared solution of Pt USNPs for an hour. The products were separated via centrifugation (12,000 rpm, 10 min) and further purified twice by deionized water and then dried in a vacuum oven at 80 °C for 8 h.

### 3.4. Characterization

The transmission electron microscopy (TEM) images were obtained on JEOL JEM-2100F instruments (Tokyo, Japan) operating at an accelerating voltage of 200 kV. For the preparation of samples for electron microscopy analyses, aliquots taken from the as-prepared Pt USNPs were dropped directly onto carbon-coated copper grids placed on a filter paper and then dried at room temperature in air. The grids were placed in a sack and steeped in alcohol for a half hour. After that, the grids were taken out and dried at room temperature in air. The structures of as-prepared products were characterized by X-Ray diffraction (XRD) (Rigaku D/Max 2550 X, Cu Kα radiation, λ = 0.154178 nm, Tokyo, Japan). The composition of Pt/Vulcan XC-72 was determined by energy dispersive X-Ray spectroscopy (EDS, Quanta FEG 250, 20 kV, FEI Company, Hillsboro, OR, USA).

### 3.5. Electrocatalytic Activity Evaluation

Electrochemical characterizations were carried out using with CHI660D and a three-electrode configuration with a saturated calomel electrode (SCE) and a platinum foil as the reference and counter electrode, respectively. The catalyst ink, which was prepared from the mixture of catalyst (2 mg), ethanol (1 mL), and Nafion solution (5%, 50 µL), was spread onto a glass carbon (GC) electrode (4 mm diameter). For all electrodes, a metal loading of 23.8 μg·cm^−2^ was used. Commercial Pt/C catalyst (30 wt %) was also tested for comparison. Cyclic voltammetry (CV) measurements were performed in 0.5 M H_2_SO_4_ solutions under a flow of N_2_ at a sweep rate of 50 mV·s^−1^. The amperometric *i*-*t* curves were obtained in 0.5 M H_2_SO_4_ + 0.5 M CH_3_OH.

## 4. Conclusions

In summary, a novel method has been successfully developed to synthesize Pt USNPs with an average size of 2.45 nm. These Pt USNPs supported on Vulcan XC-72 exhibited an enhanced activity for methanol oxidation compared to the commercial Pt/C catalyst, thus providing great potential as a promising electrocatalyst for high performance DMFCs. Our results suggest that the PVP-assisted hydrothermal method is a simple and environmentally benign route for the synthesis of Pt nanoparticles for catalysis and other applications. Significantly, our results also provide a new insight into the role played by the hydrothermal method in the synthesis of noble metal nanoparticles.
